# Role of radiation therapy in primary tonsil large B cell lymphoma: a SEER-based analysis

**DOI:** 10.1186/s13014-021-01919-x

**Published:** 2021-10-02

**Authors:** Jing Jia, Wenming Chen

**Affiliations:** Department of Hematology, Beijing Chaoyang Hospital, Capital Medical University, Beijing, 100020 China

**Keywords:** Tonsil DLBCL, Radiation, Nomogram, SEER, Survival

## Abstract

**Backgroud:**

Primary tonsil diffuse large B cell lymphoma (PT-DLBCL) is an uncommon disease entity. The role of radiation therapy (RT) in PT-DLBCL is debatable in both the pre- and post- rituximab era. The purpose of this study was to evaluate the treatment outcome and establish a prognostic model in PT-DLBCL based on the Surveillance, Epidemiology, and End Results (SEER) database.

**Materials and methods:**

Data of 1214 PT-DLBCL patients diagnosed between 1975 and 2016 were extracted from SEER 18. The effect of RT was assessed for the entire cohort and subgroups by stages using univariate, multivariate Cox regression analyses and propensity score matching (PSM).

**Results:**

The entire cohort included 1043 patients with early-stage (ES) PT-DLBCL and 171 patients with advanced-stage (AS) disease. A decreasing trend of RT utilization in the ES cohort after 2002 was observed. 47.4% of patients in ES received RT, whereas 25.1% in AS underwent RT. RT significantly improved overall survival in both univariate (*P* < 0.001) and multivariate (*P* = 0.002) analyses. PSM analysis further validated the survival advantage of RT (*P* = 0.002). A nomogram was established to predict the potential survival benefit. Subgroup analysis revealed RT was significantly associated with overall survival in ES patients of PT-DLBCL (*P* = 0.001) and in the rituximab era (*P* = 0.001) but not in those with AS disease (*P* = 0.241).

**Conclusions:**

This population-based study encloses the largest sample of PT-DLBCL to date and demonstrates a favorable survival role of RT in early stages rather than advanced stages. The established nomogram helps to identify high risk patients to improve prognosis.

## Introduction

Waldeyer’s Ring is a circular region of lymphoid tissue which consists mainly of nasopharynx, oropharynx, tonsils and the base of tongue. It accounts for more than half of non-Hodgkin’s lymphoma (NHL) in the head and neck and nearly 40–60% of these patients present as primary tonsil lymphoma (PTL) [1–3]. The most common histologic subtype of PTL is diffuse large B cell lymphoma (DLBCL) [4]. Recently, primary tonsil DLBCL (PT-DLBCL) used to be regarded as extranodal lymphoma has been reclassified as nodal lymphoma [5]. Patients with PT-DLBCL often present as a sore throat and dysphagia, with sign of tonsillar swelling and cervical adenopathy.

The majority of PT-DLBCL patients present with localized disease (stage, I/II) and radiation therapy (RT) alone reported as an effective treatment option for these patients, resulting in a 5-year overall survival (OS) of 50% [6]. However, over 40% of these patients relapsed at sites outside the primary radiation field [7]. An Indian study reported in patients with DLBCL of the tonsil, chemotherapy (CT) + RT resulted in a significantly better outcome than those treated with CT alone and the complete response (CR) and OS rate were significantly better for patients receiving an RT dose ≧ 45 Gy [8]. Whereas study from the International Extranodal Lymphoma Study Group (IELSG) showed consolidation RT did not prolong lymphoma specific survival in patients with early-stage DLBCL of Waldeyer’s ring in remission after anthracycline-containing CT [9]. Moreover, radiation at this special anatomical site of head and neck field may cause acute and chronic events that exhibit negative effect on the quality of patients’ life or survival, such as oral mucositis, dental decay, xerostomia, hypothyroidism and secondary malignancy.

To our knowledge, the introduction of anti-CD20 antibody rituximab has significantly improved the response, disease free survival and OS of patients with DLBCL since 2002 [10]. A retrospective research on the role of consolidative RT after the rituximab, cyclophosphamide, doxorubicin, vincristine, and prednisone (R-CHOP) immunochemotherapy in early-stage DLBCL of Waldeyer’s ring concluded no survival advantage of RT in these patients [11]. Based on these evidence, the necessity of applying RT in PT-DLBCL remains controversial and requires further investigation.

## Materials and methods

### Data source

This retrospective cohort study was performed using data from the Surveillance, Epidemiology, and End Results (SEER) 18 registry (1975–2016 varying) database. SEER accounts for cancer registries covering approximately 28% of the U.S. population. Based on the third edition of the International Classification of Disease for Oncology (ICD-O-3) codes for histology (9680) and topography (C09.9), we included patients histologically diagnosed as PT-DLBCL. Patients younger than 18 years old, diagnosed on autopsy or death certificate, with incomplete follow-up data or no information on disease stage were excluded. All patients enrolled were subject to CT as part of treatment.

### Study variables

The data obtained included year of diagnosis, age at diagnosis, sex, race, Ann Arbor stage, survival time, and marital status. The Ann Arbor stages were categorized into early stage (ES) for stage I/II and advanced stage (AS) for stage III/IV. Marital status was classified as married (including common law) and other (single/separated/divorced/widowed/unmarried or domestic partner).

### Construction and validation of the nomogram

The study participants were randomly allocated to two sequential cohorts: a model derivation data set (two-thirds, N = 790) and a validation data set (one-third, N = 424). Then a nomogram was established to individually predict patients’ 3-, 5-, and 10-year survival rates. The nomogram was both internally and externally validated by measuring discrimination and calibration curves. As previously described [12, 13], internal validation was carried out with bootstrap resamples, in which regression models were fitted in 1000 bootstrap replicates, drawn from the development sample. External validation was performed with the validation datasets. Concordance index (C-index) is used to measure the ability of the nomogram to discriminate between the predicted and real values in survival analysis. A C-index value of 0.5 indicates no predictive discrimination and a value of 1.0 indicated perfect separation of patients with different outcomes. Calibration plots exhibit the capability to examine how well the model-based predicted probabilities of survival agreed with the observed probabilities, and an entirely accurate nomogram would result in a plot on which predictions fall along a 45◦ diagonal line [14, 15].

### Statistical analysis

The statistical analysis was performed using tools of the SEERstat 8.3.8, R software version 3.6.3 and SPSS version 25. Survival curves were generated according to the Kaplan–Meier method and compared using the log-rank test. Prognostic factors were investigated by univariate, and multivariate Cox regression analyses. To further adjust for any potential confounders that could cause bias, a propensity score matching (PSM) accounting for all the covariates was performed. In brief, propensity scores were obtained using multivariable logistic regression predictive of treatment assignment (CT or combined modality therapy (CMT)). A ratio of 1:1 with the propensity score radius difference of 0.02 was chosen to maximize the balance between treatment groups. Survival analyses were carried out using a Cox proportional hazards model, which were used to compare the survival between the two matched groups. All statistical tests were two-sided with a significant threshold of 0.05.

## Results

### Clinical characteristics

**Fig. 1:**
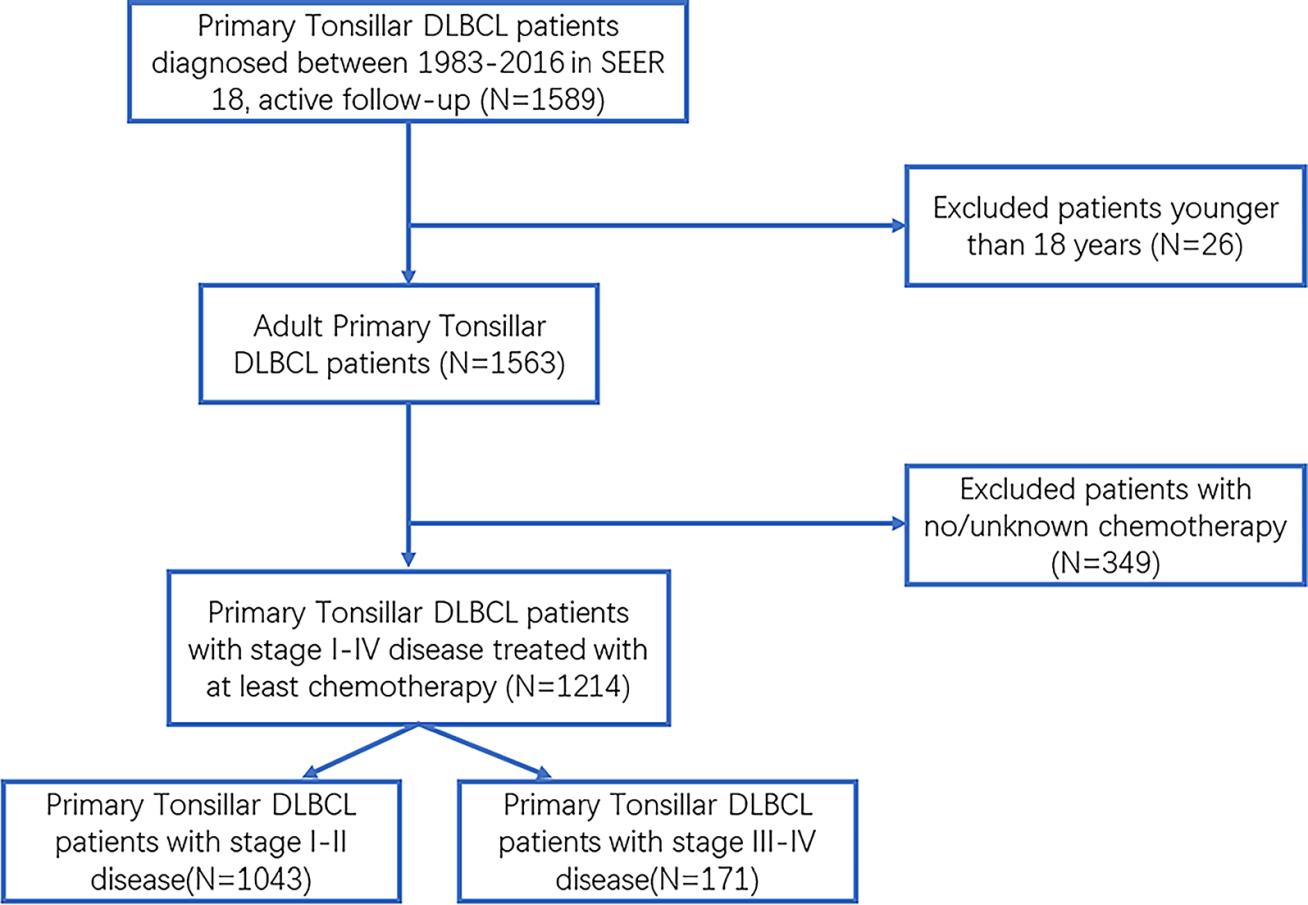
Flow chart for patient selection from SEER 18. DLBCL, diffuse large B cell lymphoma

We identified 1214 adult patients diagnosed with PT-DLBCL and treated with at least CT as part of therapeutic choice through query of SEER 18 (Fig. 1). Demographic characteristics of patients in the cohort were outlined in Table 1. The median age was 61 years (range 18–98). Patients with PT-DLBCL were more likely to be males (58.1%), white (78.2%), with an ES predominance (85.8%). The majority of patients received CT alone (55.8%). As shown in Table 1, after PSM, the imbalance between CMT and CT groups was avoided for all the included parameters.

**Table 1:** Patient Characteristics

Characteristic	Patient characteristics in raw data				Patient characteristics after propensity score matching		
	Total	Chemotherapy	Combined modality therapy	*P*	Chemotherapy	Combined modality therapy	*P*
	1214	677 (55.8%)	537 (44.2%)		455 (50.0%)	455 (50.0%)	
Age, y				0.115			0.342
18–39	134 (11.0%)	72 (10.6%)	62 (11.5%)		57 (12.5%)	53 (11.6%)	
40–64	556 (45.8%)	295 (43.6%)	261 (48.6%)		202 (44.4%)	224 (49.2%)	
65+	524 (43.2%)	310 (45.8%)	214 (39.9%)		196 (43.1%)	178 (39.1%)	
Sex				0.986			0.544
Male	705(58.1%)	393(58.1%)	312 (58.1%)		272 (59.8%)	263 (57.8%)	
Female	509(41.9%)	284(41.9%)	225 (41.9%)		183 (40.2%)	192 (42.2%)	
Year of diagnosis				< 0.001			0.653
1983–2001	327(26.9%)	148(21.9%)	179 (33.3%)		124 (27.3%)	118 (25.9%)	
2002–2016	887(73.1%)	529(78.1%)	358 (66.7%)		331 (72.7%)	337 (74.1%)	
Race				0.123			0.066
White	943(78.2%)	527(78.5%)	416 (77.8%)		369 (81.1%)	356 (78.2%)	
Black	76(6.3%)	49 (7.3%)	27 (5.0%)		31 (6.8%)	22 (4.8%)	
Other	187(15.5%)	95 (14.2%)	92 (17.2%)		55 (12.1%)	77 (16.9%)	
Stage				< 0.001			0.912
I	431 (35.5%)	213 (31.5%)	218 (40.6%)		179 (39.3%)	182 (40.0%)	
II	611 (50.3%)	336 (49.6%)	275 (51.2%)		229 (50.3%)	232 (51.0%)	
III	77 (6.3%)	58 (8.6%)	19 (3.5%)		17 (3.7%)	16 (3.5%)	
IV	95 (7.8%)	70 (10.3%)	25 (4.7%)		30 (6.6%)	25 (5.5%)	
Marital status				0.031			0.493
Married	701 (60.5%)	369 (57.7%)	332 (64.0%)		290 (63.7%)	280 (61.5%)	
Other	457 (39.5%)	270 (42.3%)	187 (36.0%)		165 (36.3%)	175 (38.5%)	

**Fig. 2:**
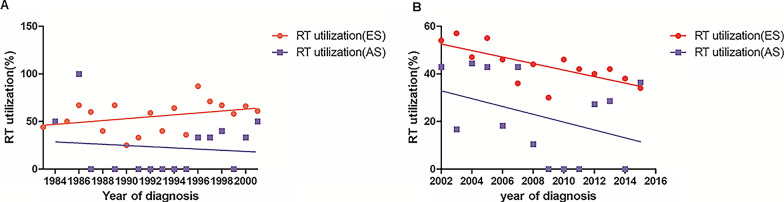
Trends of RT use in PT-DLBCL by different stages. **A** For patients diagnosed between 1983 and 2001. **B** For patients diagnosed between 2002 and 2016

The utilization situations of RT over time were displayed in Fig. 2. In pre-rituximab era, RT utilization rate by year was stable in both early ES and AS patients (slope for the best fit line = 1.004, *P* = 0.1271; slope for the best fit line = − 0.6252, *P* = 0.6928). However, RT utilization rate dramatically decreased in ES (slope = − 1.369, *P* = 0.0038) but didn’t change significantly in AS patients after 2002 (slope = − 1.648, *P* = 0.1759).

### Survival and prognostic factors

#### Univariate and multivariate cox proportional hazard analyses

**Fig. 3:**
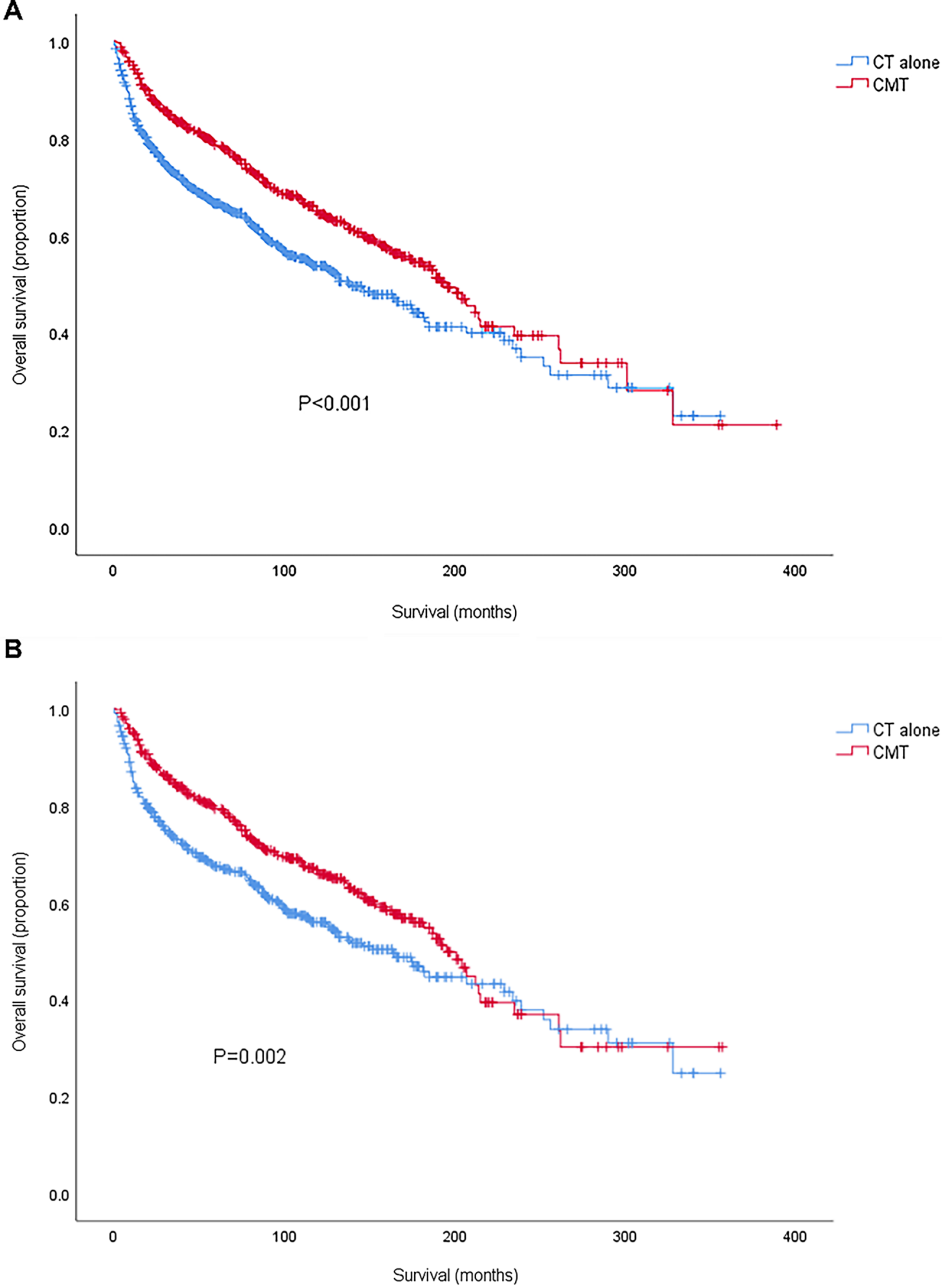
Kaplan–Meier survival curves comparing CT alone and CMT before (A) and after (B) propensity score matching. CT, chemotherapy; CMT, combined modality therapy

The estimated 5-year OS for the entire cohort was 71.8%. CMT was associated with a significantly better 5-year OS compared with CT alone: 78.4 vs. 66.6%, respectively (HR = 0.689, 95% CI 0.575–0.827, *P* < 0.001). Kaplan–Meier survival curves for CMT and CT treatment groups were depicted in Fig. 3 A. On multivariate analyses, CMT remained a favorable influence on OS (HR = 0.42, 95% CI 0.614–0.897, *P* = 0.002). Both univariate and multivariate analyses revealed a significantly worse OS for patients with older age, increasing stage, diagnosis before 2001 and marital statuses other than marriage. PSM confirmed the protective role of RT utilization for OS (HR = 0.721, 95% CI 0.585–0.889, *P* = 0.002) and Kaplan–Meier survival curves for the PSM analysis were displayed in Fig. 3B. Older age, increasing stage and diagnosis before 2001 were also independent prognostic factors of worse survival while there was no significant survival difference between different marital statuses after adjusting for the imbalance between all baseline variables (Table 2).

**Table 2:** Prognostic factors for overall survival

	Univariate		Multivariate		Propensity score cox regression	
	HR (95 %CI)	P	HR (95 %CI)	P	HR (95 %CI)	P
Treatment		< 0.001		0.002		0.002
Chemotherapy alone	Reference		Reference		Reference	
Combined modality therapy	0.689 (0.575–0.827)		0.742 (0.614–0.897)		0.721 (0.585–0.889)	
Age, y		< 0.001		< 0.001		< 0.001
18–39	Reference		Reference		Reference	
40–64	2.996 (1.755–5.113)		3.648 (2.093–6.359)		3.342 (1.794–6.223)	
65+	11.806 (6.980-19.968)		13.448 (7.792–23.210)		13.696 (7.405–25.333)	
Sex		0.081				0.774
Male	Reference				1.031 (0.836–1.272)	
Female	1.173 (0.981–1.404)					
Year of diagnosis		< 0.001		< 0.001		< 0.001
1983–2001	Reference		Reference		Reference	
2002–2016	0.658 (0.543–0.797)		0.612 (0.503–0.746)		0.630 (0.503–0.788)	
Race		0.119				0.211
White	Reference				Reference	
Black	0.786 (0.535–1.155)				0.724 (0.454–1.154)	
Other	0.785 (0.601–1.027)				0.818 (0.596–1.124)	
Stage		< 0.001		< 0.001		< 0.001
I	Reference		Reference		Reference	
II	0.943 (0.771–1.153)		0.922 (0.750–1.134)		0.976 (0.779–1.222)	
III	1.713 (1.195–2.455)		1.502 (1.042–2.164)		1.869 (1.144–3.055)	
IV	2.368 (1.775–3.161)		2.170 (1.610–2.925)		1.865 (1.276–2.723)	
Marital status		0.001		< 0.001		0.416
Married	Reference		Reference		Reference	
Other	1.347 (1.121–1.619)		1.408 (1.168–1.698)		1.093 (0.882–1.356)	

### Construction and validation of a prognostic nomogram

**Fig. 4:**
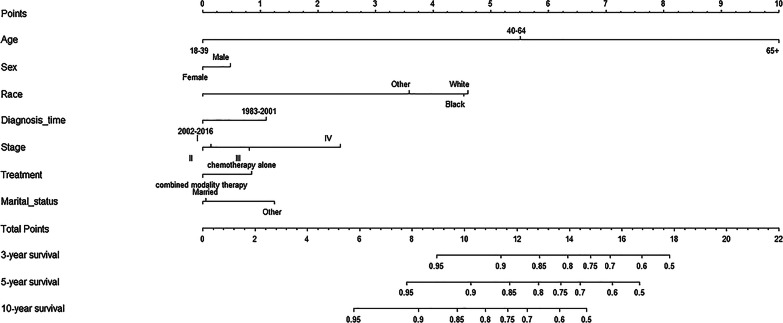
Prognostic nomogram to predict 3-, 5-, 10-year overall survival in PT-DLBCL patients

**Fig. 5:**
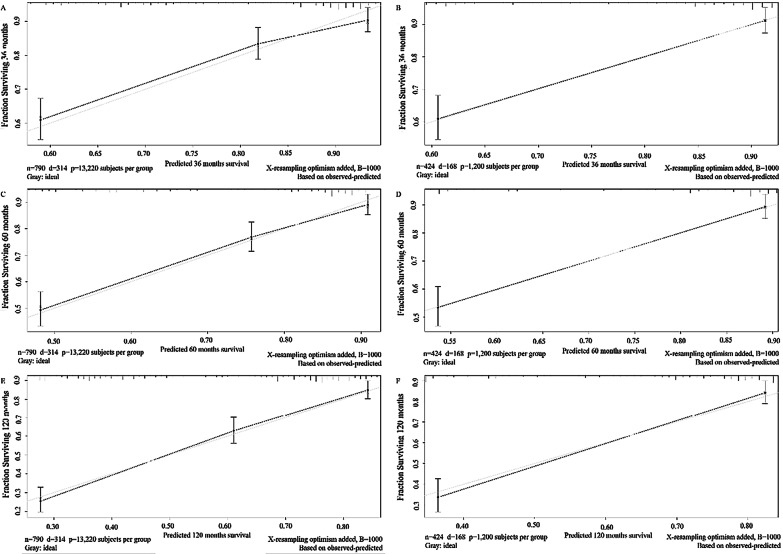
The calibration curves for predictions of overall survival in the training and validation cohorts at 3-, 5-, 10-year

A nomogram including significant indicators was developed to predict 3-, 5- and 10-year OS for PT-DLBCL (Fig. 4). The discriminative ability and predictive accuracy of the nomogram were examined using C-index and calibration plot for both training and validation cohorts. The C-index values on internal and external validations were 0.736 and 0.746, respectively, showing excellent performance in discriminate the outcome of patients with PT-DLBCL. Moreover, the data points in internal and external calibration plots fall close to the ideal line, showing high consistency between predicted and actual observed 3-, 5-, and 10-year survival for PT-DLBCL patients (Fig. 5).

### Role of RT in early-stage patients

**Fig. 6:**
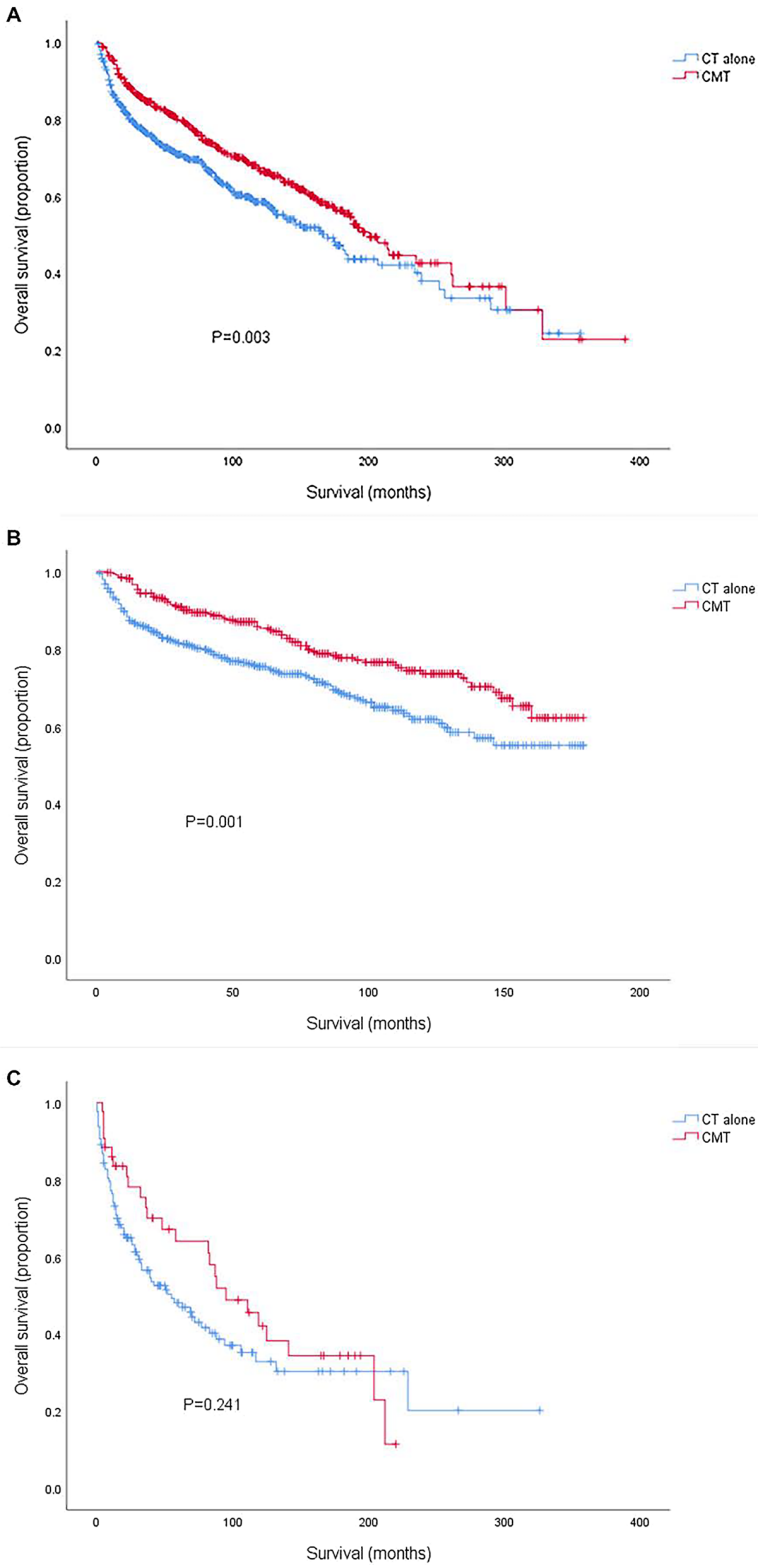
Kaplan–Meier survival curves in patients with stage I–II (**A**), stage I-II (**B**) in rituximab era and stage III-IV (**C**) comparing CT alone and CMT. CT, chemotherapy; CMT, combined modality therapy

A total of 1043 patients were diagnosed with stage I-II PT-DLBCL, with a median follow-up of 74 months (range 0–389). 47.4% of these patients received CMT. The impact of RT on OS for patients with ES disease was outlined in Fig. 6A. On univariate analysis, CMT demonstrated a prolonged OS (5-year OS = 90.8%, HR = 0.735, 95 %CI = 0.601–0.900, *P* = 0.003) compared to CT alone (5-year OS = 79.6%). In adjusted multivariate Cox model, radiotherapy, age at diagnosis, diagnosis time, race and marital status remained independent prognostic factors for OS. By PSM, imbalance in potential baseline confounders across the two treatment groups could be avoided for most patient- and treatment-related factors, except for race. CMT was still significantly associated with better OS (HR = 0.688, 95% CI = 0.549–0.862, *P* = 0.001). OS was better for patients diagnosed at younger ages and after 2002 in both groups.

We further determined the role of RT in ES patients diagnosed after 2002 indicating the rituximab era. 760 patients with ES disease were diagnosed after 2002. 43.2% of this cohort received CMT. Both univariate and multivariate analysis confirmed CMT to be correlated with better OS compared to CT alone (HR = 0.618, 95% CI = 0.463–0.826, *P* = 0.001, Fig. 6B).

### Role of RT in advanced-stage patients

171 patients presented with AS disease. The median follow-up period was 37 months, ranging from 0 to 326. A minority of patients (25.1%) in stage III-IV underwent CMT as their treatment course. Whereas, neither univariate nor multivariate analysis demonstrated any significant association between additional RT and survival benefits (5-year OS = 64.1% for CMT and 48.1% for CT alone, *P* = 0.241, Fig. 6C). Only age at diagnosis was independently associated with OS in both groups.

## Discussion

To the best of our knowledge, this is the largest population based study of PT-DLBCL using tools of PSM and individualized prediction model to clarify the role of RT in different stages of the disease. Three key points were presented: First, a decrease in utilization rate of RT was observed in patients with ES PT-DLBCL, while the proportion of RT application in AS patients remained lower and stable since 2002. Second, a clear association of chemoradiotherapy in stage I–II patients with decreased overall mortality in a large patient cohort whereas no significant difference in stage III–IV was detected, even after adjusting in multivariable or propensity score analyses. Third, even with the addition of rituximab, RT was predictive of a better outcome in patients with limited-stage PT-DLBCL.

PT-DLBCL is a distinct clinicopathologic entity with a predominance of germinal-center-like immunophenotype [16]. Histologically, the focal follicular features in PT-DLBCL suggested a pathological subgroup different from *de novo* nodal DLBCL, possibly representing follicular colonization of marginal zone B cell lymphoma or transformed follicular lymphoma [17]. The clinical picture of DLBCL of Waldeyer’s ring showed a preference for gastro-intestinal involvement [18]. A high Ki-67 index, lymphocyte count at diagnosis < 1.000/mm(3) and the Bcl-2 protein expression were reported to be negative prognostic factors in patients with PT-DLBCL [19]. The common practice for initial treatment of DLBCL is 4 to 6 cycles of R-CHOP with or without RT depending on disease stages, International Prognostic Index (IPI) risk groups and bulky diseases. However, this approach especially for PT-DLBCL remains debatable as new knowledge becomes available.

For limited DLBCL, 4 randomized trials conducted in the prerituximab era indicated supportive evidence in RT for consolidation [20–23]. Ezzat et al. reported a significantly better event free survival for combination of CT + RT in localized NHL of Waldeyer’s ring [24]. Another study found in 121 patients with PT-DLBCL (95% stage I/II), CT + RT resulted in a significantly better outcome than those treated with CT alone (10-year OS: 85.7% vs. 70.7%, *P* = 0.008). Even patients who attained CR after rituximab-naïve CT benefited significantly from consolidation RT (10-year disease free survival: 96.2% vs. 54.4%, *P* < 0.001) [8]. Consistent to these studies, a clear survival benefit of CMT in limited stage PT-DLBCL was also demonstrated in our investigation.

Whether radiation could be omitted in the rituximab era has become controversial. Several studies in the postrituximab era confirmed the benefit of RT for limited stage DLBCL with bulky disease [25–27]. In the UNFOLDER trial by the DSHNHL, patients with bulky tumor were randomized to R-CHOP with or without RT. Interim analysis showed a higher failure rate in no-RT group [28]. Our study consistently confirmed the favorable survival role of RT in patients with ES PT-DLBCL in the rituximab era. However, Guo et al. retrospectively analyzed the role of consolidation RT in patients with stage I/II DLBCL limited in Waldeyer’s ring after CR from R-CHOP. The 5-year PFS rates in CT + RT group vs. CT group were 93.3% vs. 92.5% (*P* = 0.896) and the 5-year OS rates were 96.7% vs. 94.4% (*P* = 0.649). But no bulky disease was included in this study[11]. Accordingly for limited PT-DLBCL, consolidation RT in the rituximab era should be selectively administrated to those with bulky disease. For advanced DLBCL, the role of consolidative RT to bulky disease is supported by 2 postrituximab studies and a retrospective match-pair analysis [25–27]. At this point, RT application in AS PT-DLBCL remains at the institutional discretion, but is in general used for sites that are bulky (> 5 cm), did not achieve a CR, or are adjacent to critical organs [29]. Lee et al. evaluated 19 patients with PT-DLBCL treated with CT combined with RT. The 5-year PFS rates in the CHOP + RT (> 40 Gy) group vs. R-CHOP + RT (≤ 40 Gy) group were 50% vs. 100% (*P* = 0.018) and 5-year OS rates were 66.7 %vs. 100% (*P* = 0.087) [30]. RT dose reduction may be the trend for PT-DLBCL in the rituximab era especially for favorable good responders. Guidelines from international lymphomaradiation oncology group (ILROG) recommended the radiation dose for PT-DLBCL can be 30 to 40 Gy depending on the bulk of the disease and its response to chemotherapy [31, 32]. Patients with a documented CR received RT to 30 Gy and those with a partial response and/or bulky disease received 40 Gy [33]. As to the radiation field, involved-site radiation therapy (ISRT) has been proposed which treated only the site of initial involvement incorporating computed tomography (CT) or positron emission tomography (PET)-CT based treatment planning [34, 35]. The clinical target volume (CTV) should include the involved tonsillar area with neck lymph nodes (only if involved) based on pretherapy images [33].

The decreasing utilization of RT in PT-DLBCL over the past 2 decades might be associated with the individualized treatment strategies based on PET-CT and concern of RT related toxicities. The routine practice is to administer consolidative RT to those with positive end-of-treatment PET-CT based on available evidence [36, 37]. As a result, post-chemotherapy PET-CT can better define subgroups that could benefit from RT and avoid over-treatment with RT utilization [38]. However, a residual positivity in PET-CT must be interpreted with caution since focal inflammation or necrosis of tonsil rather than persistent lymphoma could lead to false positive cases. A long time follow up on 19 patients with tonsillar lymphoma receiving CMT showed a 5 year survival of 100%, but 21% of these survivors experienced persistent xerostomia and a fatal side effect of radiation-induced sarcoma was observed [39].

There are very few prospective data in literature illustrating the treatment modality and prognostic impact of PT-DLBCL. Nationwide datasets like SEER database have strengths resting primarily on a large sample size, high completeness of survival data and representiveness of the whole patient population [40]. However, several limitations should be acknowledged in this study. First, the inherent nature of SEER determined a lack of records about many clinical, pathological and biological information, such as presence of bulky disease, IPI or some molecular markers, which were considered important prognostic variables in other studies. Second, chemotherapy regimens and radiation doses were unclear. We supposed most patients diagnosed after 2002 might received rituximab therapy.

In conclusion, this study indicates a favorable role of RT on OS in patients with stage I-II PT-DLBCL but not in patients with AS PT-DLBCL. The nomogram will help clinicians to identify high risk patients to choose optimal treatments.

## Data Availability

The data is available on the Surveillance, Epidemiology, and End Results (SEER, http://seer.cancer.gov) database.

## Code availability

Code used for the analysis of data in this study is available from the corresponding author on reasonable request.
